# Influence of head size on the development of metallic wear and on the characteristics of carbon layers in metal-on-metal hip joints

**DOI:** 10.3109/17453670902988394

**Published:** 2009-06-01

**Authors:** Volker Braunstein, Christoph M Sprecher, Markus A Wimmer, Stefan Milz, Georg Taeger

**Affiliations:** ^1^AO Research Institute, AO FoundationDavosSwitzerland; ^2^Department of Traumatology and Orthopedic Surgery, Ludwig-Maximilians-UniversityMunichGermany; ^3^Department of Orthopedic Surgery, Rush University Medical CenterChicago, ILUSA; ^4^Department of Trauma Surgery, University HospitalEssenGermany

## Abstract

**Background and purpose** Particles originating from the articulating surfaces of hip endoprostheses often induce an inflammatory response, which can be related to implant failure. We therefore analyzed the metal content in capsular tissue from 44 McKee-Farrar metal-on-metal hip prostheses (with 3 different head sizes) and we also analyzed the morphological structure of layers located on articulating surfaces.

**Methods** Atomic absorption spectrometry (AAS) was used to analyze the metal content in capsular tissue. Visually detectable carbon layers located on the articulating surfaces were evaluated using scanning electron microscopy (SEM), energy-dispersive Xray spectroscopy (EDX), and X-ray photoelectron spectroscopy (XPS).

**Results** Metallic debris was detected in all capsular tissue samples but no statistically significant differences in metal content were found in relation to implant head size. The morphological characteristics of the different layer zones allowed an exact analysis of contact and non-contact areas. Furthermore, surface layers appear to have a protective function because they can prevent sharp-edged particles from damaging the prostheses surface.

**Interpretation** The implant head size does not appear to influence the amount of metallic debris. The layers obviously act like a lubricating agent because the protection function does not occur in regions without layers where the metal surface often shows numerous scratches. As layers are not generated immediately after the implantation of hip prostheses, these findings may at least partially explain the high amount of wear early after implantation.

## Introduction

One of the strongest growing branches in total hip arthroplasty is surface replacement with metal-on-metal components. This is based on excellent clinical results from several retrospective reports (Amstutz et al. 2007, Berend and Bertrand 2007). Components used for surface replacement consist of large-diameter femoral heads and corresponding acetabular cups. Large diameters (> 32 mm) are reported to be favorable with respect to reduced impingement and dislocations as well as to low wear rates related to the reduced range of motion between head and socket (Dowson et al. 2004). However, Wimmer et al. (2006) reported about so-called “stick phenomena” (a synonym for highest friction peaks) between the femoral and acetabular surfaces of metal-on-metal components greater than 32 mm. Investigation of the bearing surfaces of retrieved implants revealed stick phenomena in more than 80% of all surfaces examined. These findings were consistent with simultaneously conducted in vitro testing results of large-diameter implant components. It was demonstrated that these stick phenomena lead to a substantial increase in static friction between the bearing surfaces and it was concluded that this increase in friction may cause high tangential shear stress at the bone-implant interface, which could adversely affect the outcome of large-diameter metal-on-metal surfaces on a long-term basis.

Wear is usually seen as another major issue in metal-on-metal bearings. Even so, metal-on-metal bearings have been reported to show 20–100 times lower wear rates than polyethylene-on-metal bearings and even substantially lower rates than polyethylene-on-ceramic bearings (Brown et al. 2007). However, the biocompatibility of metal-on-metal joints is controversial, especially in terms of hypersensitivity, allergies, or even toxicity (Campbell et al. 2004). Some authors assume that the extent of metallic wear is less than wear from polyethylene, resulting in higher tissue concentration of alloy components and in more severe inflammatory or even toxic alteration of the surrounding tissue (Cobb et al. 2006). However, previous investigations have clearly demonstrated that wear from metal-on-metal bearings derives from surface fatigue within a nanocrystalline layer (Buscher et al. 2005). This could be considered as a strong reason why metal-on-metal bearings are linked to elevated serum levels of alloy components but nevertheless do perform well clinically with respect to overall production of wear and frequency of aseptic loosening.

Another issue that is important regarding large-diameter implants is the question of surface layers, which were described in experimental simulator tests with metal-on-metal bearings (Wimmer et al. 2001). Carbon layers on bearing surfaces are generated by tribochemical reactions. We assume that such layers, located at articulating parts of the bearing surfaces, can act as lubricant material in large-diameter implants.

Based on the knowledge gained from previous investigations on metal-on-metal bearings, this study was conducted to investigate tribochemical effects, which occur in large-diameter metal-on-metal implants. To compare different surface diameters, retrieved first-generation metal-on-metal hip implants with different head sizes (35 mm, 39 mm, and 41.5 mm) were investigated. We concentrated on the local amount of wear particles by the metal-on-metal bearings and also the topography of surface layers.

## Material and methods

### Implant and tissue retrieval

The collection of implants consisted of 44 McKee-Farrar metal-on-metal hip prostheses, which were made of cobalt-chromium alloy according to ISO 5832-4. The implants varied regarding the diameter of the head and corresponding acetabular component, with 26 implants having 35 mm diameter, 5 implants having 39 mm, and 13 implants having 41.5 mm diameter. All prostheses were implanted and explanted by the same surgeon. Primary arthroplasty for degenerative coxarthrosis was the only implantation criterion while aseptic loosening was the only precondition for inclusion in the study. Furthermore, all patients included in this study had a normal ability to walk after implantation of the prosthesis. At the time of implantation, patients were on average 62 (39–82) years old. Implants were revised mean 12 (1.1–22) years after implantation for aseptic loosening of the stem or the cup, or both components. The average time of function was 10 (1.1–22) years (SD 6.4) for 35-mm heads, 16 (11–20) years (SD 3.6) for 39-mm heads, and 13 (1.1–22) years (SD 6.8) for 41.5-mm heads. These differences were not statistically significant (p = 0.2, Kruskal-Wallis test with one-way analysis of variance on ranks).

During the removal of the prostheses, 10 grams of the anterior-lateral and the dorsolateral part of the capsule tissue was removed and fixed in 20 mL formaldehyde for further investigation using atomic absorption spectrometry (AAS). As the joint capsule was the only tissue removed, no reference tissue (without any contact with the prothesis) could be removed. Care was taken to avoid artificial damage of the bearing surfaces while removing the prothesis components. After removal, the prostheses were packed without damaging the surfaces.

### Atomic absorption spectrometry (AAS)

Tissue samples were processed for AAS, which is the most sensitive and specific technique for determining tissue concentrations of cobalt (Co), chromium (Cr), nickel (Ni), and molybdenum (Mo), all of them being alloy components. Tissue samples of at least 10 g were stored in Falcon tubes in 5 mL formaldehyde. After adding nitric acid and hydrogen, the compound was solubilized by boiling and then evaporated and excited using a flame (Verbanac 1997). Using nitric acid, the zero point of the photometer was defined before starting the measurements. Afterwards, the straight calibration line of each element was identified using the pure substance of each element. A minimum of 4 measurements were performed for establishing the straight calibration line. The metal content of the sample probes was calculated in μg metal per g of retrieved capsular tissue.

### Statistics

The Mann-Whitney rank sum test was used to evaluate differences in the total metal content for small-diameter implants (35 mm), medium-diameter implants (39 mm), and large-diameter implants (41.5 mm). In addition, the metal content of the tissue samples was related to the time of functioning until revision (μg metal per g capsular tissue per year of function in situ). Regression analysis was then performed for the metal content of small-, medium-, and large-diameter components to investigate a possible relationship between the time of function and the amount of wear. The same was done for the overall metal content. Based on the methodology of mass spectroscopy, all kinds of metallic contamination—irrespective of whether this was derived from wear particles or from corrosion products—were detected by this investigation.

### Scanning electron microscopy (SEM)

The implant surfaces were cleaned in an ultrasound bath in methanol for 5 min to allow analysis of the prostheses with scanning electron microscopy (SEM), energy-dispersive Xray spectroscopy (EDX), and X-ray photoelectron spectroscopy (XPS). Samples with visible surface layers were cut into several smaller pieces using an angle grinder. During the cutting process the samples were cooled with deionised water. SEM was used in two modes. By detecting secondary electrons (SE mode), which are reflected from the surface due to excitation by the primary electron beam, evaluation of morphological details of the surfaces was achieved. In addition, back-scattered electrons (BSE mode) were used to detect differences in material density. To prevent artefacts, all samples were analyzed uncoated.

### EDX, XPS, and protein assay

Energy-dispersive X-ray spectroscopy (EDX, measured with Model 6816; Oxford Instruments, High Wycombe, UK) was performed at 5 keV and 10 keV in order to detect amounts of carbon, nitrogen, and oxygen quantitatively.

X-ray photoelectron spectroscopy (XPS, measured with a PHI Quantum 2000 photoelectron spectrometer; Physical Electronics Inc., Eden Prairie, MN), a quantitative spectroscopic technique, was used to measure the concentration and the chemical status of elements on the sample surfaces. The area of analysis was determined by light microscopy. With XPS it was possible to analyze the thickness and the composition of the different layers. Thus, the samples were exposed to a monochromatized X-ray (A1 Kα = 1486.6 eV) X-ray beam with 10, 20, 50, or 100 μm of lateral resolution. A hemisperical electron energy analyzer equipped with a channel plate and a position-sensitive detector was used to analyze emitted photoelectrons. The take-off angle of the electrons was 45°. The total enery resolution of the analyzer was 1.70eV or 1.04 eV. During analysis, the residual background pressure inside the spectrometer was better than 2 × 10-9. The binding enery scale was calibrated for Au-4f electrons at 84.0 eV. Photoelectron peak areas after Shirley background subtraction and the built-in PHI sensitivity factors for the calculation were used to evaluate the concentrations of elements (given in atomic per cent, normalized to a total of 100). Using argon ion sputtering, several depth profiles were evaluated.

Finally, the layers were scraped from the articulating surface under microscopic control to screen them for protein structure. The samples were lyophilized, suspended in phosphate-buffered saline, and treated with ultrasound to increase solubility. Using the BioRad protein assay (BioRad, Hercules, CA), which is based on the Bradford dye-binding method (Bradford 1976), the amount of dissolved proteins was determined. A further sample without any organic material was used as a blind sample.

## Results

### Atomic absorption spectrometry

**Figure 1.**
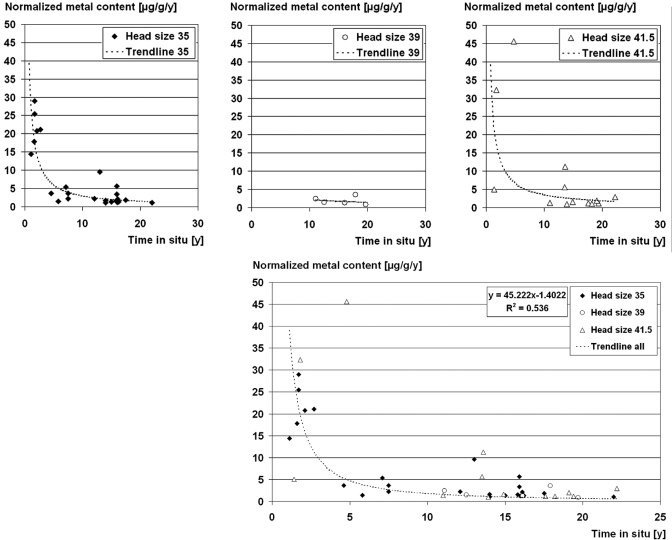
Regression analyses of metal content (μg metal per g capsular tissue per year in situ) when plotted against time in situ for individual head sizes (35 mm, 39 mm, and 41.5 mm) and for all head sizes. The resulting trend lines were approximately parallel for each head size.

In capsular tissue, the total metal content was on average 7.2 (SD 9.0) μg metal/g capsular tissue/year in situ for head size 35, 1.5 (SD 1.5) μg/g/year for head size 39, and 8.3 (SD 14) μg/g/year for head size 41.5. No statistically significant difference was found in total metal content between head sizes 35 and 41.5 (p = 0.6, Mann-Whitney rank sum test). Because of the small number, samples of head size 39 were not included in the statistical analysis. To investigate the high standard deviation values further, the metal content of all tissue samples was normalized to the time in situ. The relationship between the metal content and the time in situ of the prosthesis was evaluated using power regression. The overall regression coefficient (R2) for all head sizes was 0.536 (head size 35: R2 = 0.643, p < 0.01; head size 39: R2 = 0.171, p = 0.8; head size 41.5: R2 = 0.407, p = 0.01). The resulting trend lines were approximately parallel for each head size (Figure 1). Overall normalized metal content was higher (p < 0.001, Mann-Whitney rank sum test) within the first 3 years (20.7 μg/g/year; SD 8.6) compared to the rest of the time course (3.6 μg/g/year; SD 7.9). Interestingly, the cobalt chromium ratio of 3:1 in the alloy was inverted in all tissue samples to a ratio of 1:4.

### SEM

**Figure 2.**
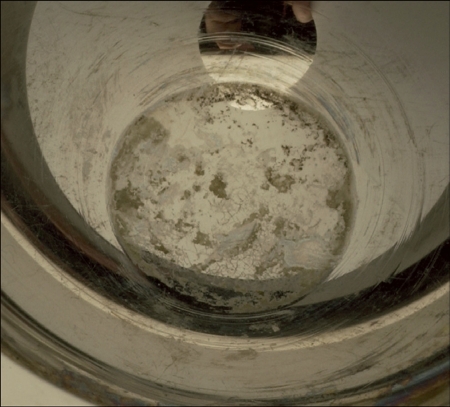
Explanted McKee-Farrar acetabular component (size 41.5 mm). All explanted prostheses showed layers that were visible to the naked eye. The morphology of the layers was inhomogeneous. Samples were cut into several smaller pieces for further evaluation.

**Figure 3.**
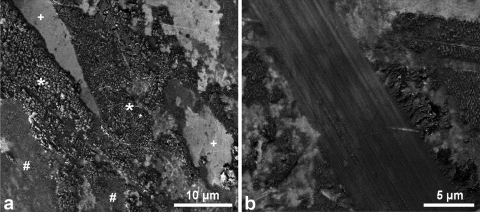
SEM images. All explanted prostheses showed layers. The morphology of these layers was inhomogeneous. a. SE mode; zones of thick layers (*) were found regions surrounded by thin layers (#) and areas without layers (+). The surface conditions of thin layers were rather smooth while thick zones showed a rough and squamous surface with cracks. The borders of thick areas were sharply defined while thin layers had rather diffuse borders. b. SE mode; smearing of thick layers without any damage to the surface. The metal surface was protected (from getting scratched) by the layer.

*Layers.* All explanted prostheses showed layers that were visible to the naked eye (Figure 2). The morphology of the layers was inhomogeneous. Using the SE mode, areas without layers and without damage were found between areas with thin layers. Zones of thick layers were found in regions surrounded by thin layers. The surface conditions of thin layers were rather smooth, while thick zones showed a rough and squamous surface with cracks. The borders of thick areas were sharply defined, while thin layers had rather diffuse borders (Figure 3a). Areas without layers and without damage presented no appearance of layer ablation because the borders of these areas had no sharp-edged or spiky configuration.

*Scratches.* Single scratches and groups of multiple scratches, which were always in parallel alignment, were found in various directions. The common characteristic was that the underlying metal surface appeared damaged in SE mode. In regions with layer formation, signs of smearing were found. Here, the SEM image (SE mode) revealed that the underlying metal surface was obviously not damaged (Figure 3b) because it appeared to be completely covered by the layer material. Layers within scratches were found in areas otherwise devoid of any other layers. Higher resolution showed that layers in scratches were composite materials built of small particles that formed agglomerates.

**Figure 4.**
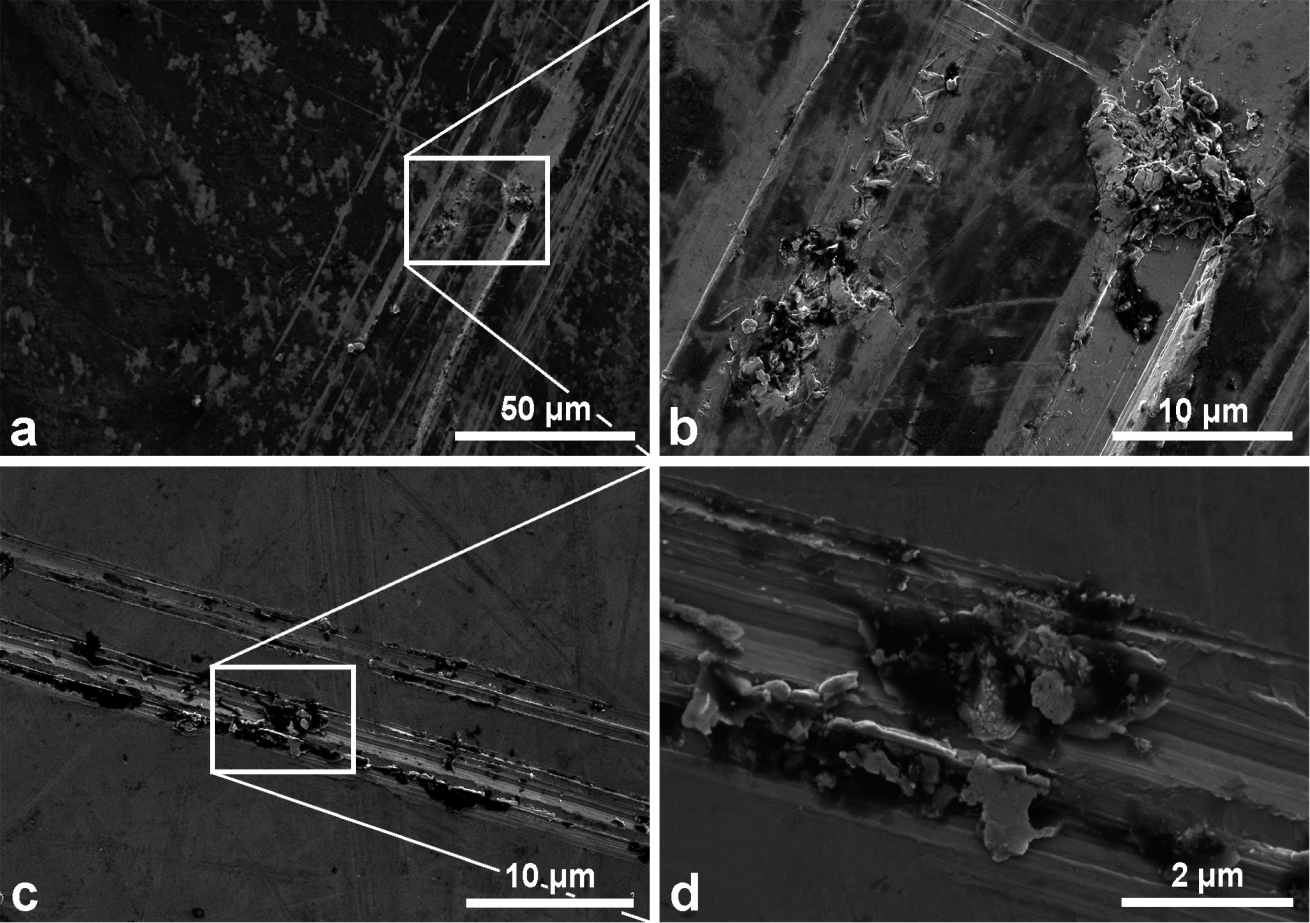
SEM images. a. SE mode; composite of particles resulting in agglomerates. b. Higher magnification of panel A: most agglomerates were found within the grooves (i.e. the deepest parts) of the scratches. c. BSE mode; bright and sharp-edged and dark and diffuse limited [unclear] particles. d. Higher magnification of panel C: the bright and sharp-edged particles are compacted in dark and diffuse limited particles.

*Agglomorates.* The agglomerates were composed of particles sized from 0.3 μm to 3.5 μm. Most agglomerates were found within the grooves (i.e. the deepest parts) of the scratches (Figure 4A and B). Using the BSE mode, two types of particles were found at higher magnification. The first type was dark and had diffuse borders; the second type was bright and sharp-edged (Figure 4C and D). The bright and sharp-edged particles were usually found within the compacted dark and diffuse-edged material. To define the origin of these particles, energy-dispersive X-ray spectroscopy (EDX) was performed.

No differences could be seen between heads and cups regarding the alignment and the size of layers, scratches, smearings, and agglomerates. Furthermore, no correlation could be found between layers, scratches, smearings, or agglomerates and the orientation of the implants in vivo.

### EDX, XPS, and protein assay

The EDX analyses of the different particle types showed that the main components of bright and sharp-edged particles were chrome and cobalt, which could only originate from the prosthesis material. The carbon-rich dark and diffuse-bordered particles contained oxygen and traces of magnesium, phosphorus, nitrogen, calcium, sodium, and chlorine.

The EDX intensity ratio of carbon and cobalt was 1:4 for bright and sharp-edged particles, which suggested that the main origin of these particles was the prosthesis material. Dark and diffusely limited particles, on the other hand, had an EDX intensity ratio for carbon and cobalt of approximately 1:0.7. Consequently, the origin of this particle type was deemed to be mainly organic. While the surface analyses of the particles were performed with an acceleration voltage of 5 keV, further analyses with an acceleration voltage of 10 keV were done to reach the underlying transition zone between the layer and the prosthesis metal. Even in regions without cobalt-chrome particles on the layer's surface, higher amounts of cobalt and chrome were found in the deep zone of the layer. As the EDX method works with cubature, exact analysis of the thickness and evaluation of the components in different layer sections was not possible. This was why XPS analysis was performed.

**Figure 5.**
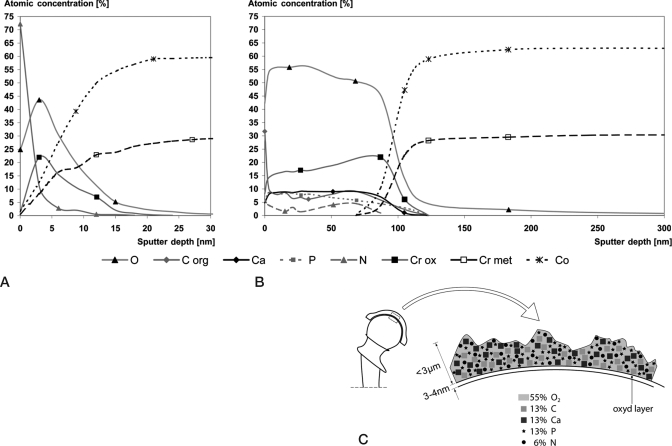
A and B. Exemplary XPS result diagrams of two different sputter depths. C. Graphical demonstration of the overall results of XPS analysis: A 3–4-nm thin oxide film was located directly on the prosthesis surface. On top of the oxide film, a second layer with a thickness of up to 3 μm and composed of different elements was detected.

XPS analysis was based on several high-resolution depth profiles. Figure 5 (panels A and B) shows two examples for two different sputtering depths. These two examples are not representative of the overall results. The overall results of the XPS analyses are shown in Figure 5C. A 3–4-nm thin oxide film was located directly on the prosthesis surface. On top of that oxide film, a second layer with a thickness of up to 3μm and composed of different elements was detected. The elements detected here were oxygen (55%), carbon (13%), calcium (13%), phosphorus (13%), and nitrogen (6%) (Figure 5C). Regarding the proportions of these components, the analyses showed minor in-depth and major lateral variations. The reason for the lateral variations is clearly related to the XPS spot-diameter of 50 μm, which was larger than the expansion of most layers themselves.

Finally, the layers that were scraped off the articulating surface were analyzed using the BioRad protein assay. The high protein concentration confirmed the organic origin of the surface layers. The blind sample did not show any proteins.

## Discussion

One of the main reasons for aseptic loosening of hip prostheses is osteolysis induced by particles (Ren et al. 2006). Larger head sizes lead to higher volumetric wear rates in polyethylene surfaces (Eggli et al. 2002). However, higher revision rates for larger head sizes have only been reported in one long-term follow-up study (Tarasevicius et al. 2006). To date, there has been no evidence from clinical studies that metal-on-metal total hip arthroplasty with large-sized heads leads to higher revision rates due to aseptic loosening, either in the first generation or in the second generation of contemporary metal-on-metal implants. The high rate of initial wear, generated in the first years after implantation and mainly caused by the “run-in period” (Dowling et al. 1978), has no immediate effect on aseptic loosening.

### Atomic absorption spectrometry (AAS)

In our study on metal-on-metal bearings, in contrast to polyethylene-on-metal bearings (Eggli et al. 2002), the head size did not have an influence on the amount of local wear in the surrounding tissue and was not related to the time in situ. In revision arthroplasty within the first 3 years, the normalized metal content was higher than in the other cases. Because aseptic loosening was the only indication for revision arthroplasty in our study, the correlation between the amount of wear and the time in situ is not surprising. Also, the “run in period” is a common effect observed in all metal-on-metal hip prostheses (Anissian et al. 2001). However, factors such as insufficient osseointegration (Maezawa et al. 2006), migration (Beaule et al. 2005), or metal hypersensitivity leading to an adaptive immune response can be additional reasons for early aseptic loosening (Willert et al. 2005). Widespread dissemination of particles in different regions of the body has been reported after total hip arthroplasty (Peoc'h et al. 1996). Although the resulting systemic effects are not preferable, the evacuation mechanism of particles could be a reason for lower particle concentration in the neighborhood of the implant, resulting in a lower rate of early aseptic loosening.

In contrast to the prostheses alloy dissemination of cobalt and chrome, the concentration of chrome was higher than that of cobalt in the capsular tissue (Tager 1976). A reason for the relatively high concentration of chrome in the capsular tissue may be found in the relatively long half-life (of disappearance from soft tissue) of chrome, which is 600 days, in contrast to the half-life of cobalt, which is only 9 days. Another reason for the relatively high concentration of chrome might be the corrosion in the surrounding of carbides (Ungethum et al. 1984) followed by loss of chrome, which deposits in the capsular tissue.

### SEM

Modifications of prosthetic surfaces follow the 4 basic types of wear mechanisms: adhesion, abrasion, surface fatigue, and tribochemical reactions (Dorinson and Ludema 1985). Each mechanism generates a worn surface with characteristic appearance, and often several of them occur together.

Deviations in shape of prosthesis components are caused by production inaccuracies (Ungethum et al. 1974) or by material abrasion (Tager et al. 1997), the latter because prosthesis components are not uniformly stressed during normal gait (Ramamurti et al. 1996). Thus, contact areas and non-contact areas have to be distinguished.

Tribochemical reactions induce protein layers that adhere to oxide layers that normally form on metal surfaces (Wimmer et al. 2006). In our study, the surfaces analyzed did not exhibit any detectable periodicity in the arrangement of thick layers, thin layers, and areas without layers. One reason for this observation may be related to the fact that completely congruent prostheses cannot be manufactured and that the degree of asphericity in new McKee-Farrar prostheses is reported to be around 3 μm (McCalden et al. 1995). In our opinion, the incidence of the different layer types is associated with the frequency of contact between the respective surface areas. Thus, direct contact between the prosthesis components did not happen in the region of thick layers after the deposit of the layer. Infrequent contacts between the load-bearing surfaces (i.e. in the extremes of joint movement) caused areas with thin layers. In regions without layers, the high frequency of component contact prevented the deposition of layers. In our opinion, the type of layer found in a certain region of the articulating surface can give information about the type and frequency of direct contact between the prosthesis components.

Scratches are generated by direct contact between prosthesis components or from particles. The size of the particles and the specific loading area define the extent of wear (Walker and Erkmann 1972). Thus, accumulations of scratches are found in zones of frequent contact or in areas with free particles. However, regarding the entire surface of prostheses, the alignment of single or groups of scratches was irregular. Smearing of layers is caused by the same mechanism as scratches, but without damaging the surface. In our opinion, the metal surface was protected from getting scratched by the layers in these cases. No differences could be found between heads and cups regarding the alignment and the size of layers, scratches, smearings, and agglomerates, and no correlation was found between layers, scratches, smearings, or agglomerates and the in vivo orientation of the implants. Thus, any prediction regarding differences of wear in different regions of a prosthesis seems to be impossible.

Discontinuation of scratches can be explained by the discontinuation of the cobalt alloy and the resulting “polishing effect” (McKellop et al. 1997). On the other hand, carbides are components of metal-on-metal prosthesis alloy (Streicher 1995). The difference between the hard carbides and the softer, surrounding cobalt alloy may be a reason for the interruptions of scratches.

Compacted and squeezed parts of layers and particles released from the prosthesis surfaces were components of the agglomerates seen. Wear is the result of local overstraining of material strength. The disconnection of material at the prosthesis surface causes particles (Streicher 1995). Movement between the prosthesis components causes the motion of these particles. In regions without layers, moving particles induce scratches. In regions with layers, particles in motion cause the compression of layers—resulting in agglomerates. A similar effect like the above-mentioned surface protection by layers is imaginable in the case of agglomerates. Because of the soft surrounding material, the sharp-edged particles are wrapped and thus inactivated.

### EDX, XPS, and protein assay

The different properties of surface layers of prostheses, such as solid lubrication and adhesion prevention, are well described (Wimmer et al. 2001). Using EDX, the different components of agglomerates identified by SEM were analyzed. Bright, sharp-edged, and chrome-and cobalt-rich particles originating from the prosthesis material were surrounded by diffuse limited carbon-rich particles originating from the surface layers. These findings confirm the assumption that layers have the capacity to “defuse” or counteract the effects of sharp-edged particles, resulting in protection of the prosthesis surface.

XPS and the protein assay confirmed the organic origin of the surface layers. Local contact stress induces the increase in temperature up to 60°C. Denaturation of proteins that are a normal part of synovial fluid is followed by this increase in temperature (Kuhlmann-Wilsdorf 1988).

### Conclusion

Regarding the amount of wear, the head sizes of prostheses had no significant influence. Consequently, the time in situ of the prosthesis was not influenced by the head size. All cases of early aseptic loosening were coupled to a high amount of particles. Although factors such as osseointegration, migration, or metal hypersensitivity might lead to early aseptic loosening, the amount of particles generated (resulting in a high metal content in the surrounding tissue) is still a crucial factor for the survival rate of hip prostheses.

The extent and the alignment of wear and debris are directly linked to factors such as prostheses material and design, technique of implantation, and activities of patients resulting in the extent of motion. The individual needs of different patients are variable. The alignment of layers, scratches, and agglomerates analyzed in our study enables an exact analysis of contact and non-contact areas on prosthesis surfaces after removal. No regularity in the configuration of layers, scratches, and agglomerates was found. This may be related to the individual demands of each patient. Consequently, a prognosis for the occurrence of wear is not possible. Even so, analysis of the amount and the alignment of layers, scratches, and agglomerates can give an idea of how to modify the design and the material a prosthesis.

Furthermore, surface layers—which originate from syno-vial fluid—have the important function of protection. Within such layers, sharp-edged particles produce no damage to the prostheses material. Thus, these layers have the capacity to counteract the damaging effects of such particles.
